# Effects of a Supervised Nordic Walking Program on Obese Adults with and without Type 2 Diabetes: The C.U.R.I.A.Mo. Centre Experience

**DOI:** 10.3390/jfmk5030062

**Published:** 2020-08-07

**Authors:** Roberto Pippi, Andrea Di Blasio, Cristina Aiello, Carmine Fanelli, Valentina Bullo, Stefano Gobbo, Lucia Cugusi, Marco Bergamin

**Affiliations:** 1 Healthy Lifestyle Institute, C.U.R.I.A.Mo (Centro Universitario Ricerca Interdipartimentale Attività Motoria), University of Perugia, Via G. Bambagioni, 19 06126 Perugia, Italy; roberto.pippi@unipg.it (R.P.); cristina.aiello@hotmail.com (C.A.); carmine.fanelli@unipg.it (C.F.); 2 Department of Medicine and Aging Sciences, ‘G. d’Annunzio’ University of Chieti-Pescara, 66100 Chieti Scalo, Italy; andiblasio@gmail.com; 3 Department of Medicine, Sport and Exercise Medicine Division, University of Padova, Via Giustiniani 2, 35128 Padova, Italy; valentina.bullo@unipd.it (V.B.); marco.bergamin@unipd.it (M.B.); 4 Department of Biomedical Sciences, University of Sassari, 07100 Sassari, Italy; lucia.cugusi@uniss.it

**Keywords:** Nordic walking, obesity, type 2 diabetes, cardiometabolic fitness

## Abstract

Exercise is a convenient non-medical intervention, commonly recommended in metabolic syndrome and type 2 diabetes (DM2) managements. Aerobic exercise and aerobic circuit training have been shown to be able to reduce the risk of developing DM2-related complications. Growing literature proves the usefulness of Nordic walking as exercise therapy in different disease populations, therefore it has a conceivable use in DM2 management. Aims of this study were to analyze and report the effects of two different supervised exercises (gym-based exercise and Nordic walking) on anthropometric profile, blood pressure values, blood chemistry and fitness variables in obese individuals with and without DM2. In this study, 108 obese adults (aged 45–65 years), with or without DM2, were recruited and allocated into one of four subgroups: (1) Gym-based exercise program (*n* = 49) or (2) Nordic walking program (*n* = 37) for obese adults; (3) Gym-based exercise program (*n* = 10) or (4) Nordic walking program (*n* = 12) for obese adults with DM2. In all exercise subgroups, statistically significant improvements in body weight, body mass index, fat mass index, muscular flexibility and maximal oxygen uptake (VO_2_ max) were observed. Moreover, a higher percentage of adherence to the gym-based program compared to Nordic walking was recorded. Our findings showed that, notwithstanding the lower adherence, a supervised Nordic walk is effective as a conventional gym-based program to improve body weight control, body composition parameters, muscular flexibility and VO_2_ max levels in obese adults with and without type 2 diabetes.

## 1. Introduction

Physical inactivity is one of the most common risk factors that increase the risk of developing relevant non-communicable diseases (e.g., type 2 diabetes, DM2) and their related risk of mortality [1]. In 2019, the International Diabetes Federation (IDF) estimated that approximately 463 million people have diabetes (type 1 and 2, diagnosed and undiagnosed) worldwide [2]. To counteract and contain this phenomenon, worldwide actions aimed to promote specific health prevention interventions. These interventions aim to reduce cardiometabolic risk factors, combining a balanced diet with an adequate level of weekly physical activity (PA), and drugs consumption where necessary.

Exercise is an effective non-medical intervention for the management of metabolic syndrome and DM2 [3,4]. In fact, aerobic exercise is the most studied type of exercise and most prescribed in people with common non-communicable chronic diseases, and it has shown to elicit beneficial effects in metabolic, Hb1Ac, body weight and insulin resistance control, also improving fat distribution and microcirculatory function [5,6], with a major effect achieved when combined with resistance training [7,8]. Moreover, aerobic exercise is able to lower the risk of DM2-related complications, such as diabetic nephropathy, retinopathy and neuropathy [9].

Walking is one of the most common physical activities, rarely associated with physical injuries due to its executive characteristics [10]. It can be performed in different environments with no need for particular equipment, overcoming some common barriers such as lack of time, low fitness level, and shortage of money. Walking is able to increase insulin sensitivity and reduces many cardiovascular risk factors, such as hypertension, dyslipidemia and fat mass accumulation [11,12,13].

Therefore, studying the effects of a particular type of walking practice, such as Nordic walking (NW), could be useful and could help to set an exercise prescription for NW practitioners. NW is an easy-to-learn activity that can be practiced anywhere and only requires the practitioner to be equipped with specific poles. NW involves upper and lower limbs simultaneously, with an increase of approximately 23% in energy expenditure compared to common walking activity [14,15], potentially ensuring major positive physiological effects linked with the prolonged and contemporaneous use of big muscle masses. Several studies showed the beneficial effects of NW in different disease populations [15,16,17,18,19], but only a few studies have investigated the cardiometabolic effects of NW in individuals with obesity and DM2 [20,21,22,23]. So, the aims of our study were to analyze and report the cardiometabolic effects following a multidisciplinary intervention performed at the Centro Universitario Ricerca Interdipartimentale Attività Motoria (C.U.R.I.A.Mo.) center, which included two different forms of supervised exercise programs (NW outdoor and exercise indoor) for individuals with obesity and DM2. The cardiometabolic effects of NW activity were compared with those aroused from a conventional exercise-based intervention performed indoor (gym-based exercise, GYM), which combined aerobic and resistance exercises.

## 2. Materials and Methods

### 2.1. Participants

**Figure 1:**
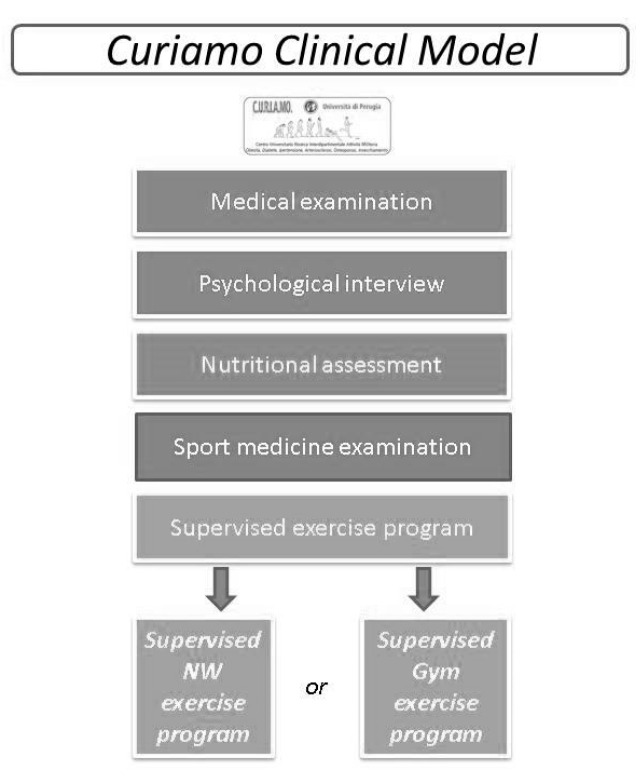
The “Centro Universitario Ricerca Interdipartimentale Attività Motoria” clinical model (De Feo et al., JEI 2011 [24]).

From 2010 to 2014, a total sample of 108 obese adults (73 women and 35 men) with and without DM2 (mean age of 56.44 ± 5.94 years) were recruited at the C.U.R.I.A.Mo. center to follow an intensive and multidisciplinary intervention protocol (Figure 1), comprising Nordic walking (NW) and a gym-based exercise program (GYM).

Inclusion criteria were: age between 45 and 65 years and a body mass index (BMI) ≥ 30 kg/m^2^. The exclusion criteria were: presence of musculoskeletal disorders or other clinical conditions that could seriously reduce life expectancy or their ability to participate in the study. Women were 45–65 years old (mean age = 56.37), but no data about menopause were collected, and the statistical analyses conducted, revealed no age effects on the considered variables both at T0 and T1.

In accordance with the subjects’ clinical conditions, which were evaluated during the first medical examination (obese individuals with or without DM2), and the different forms of exercise programs proposed (NW or GYM), participants were allocated into 1 of the 4 subgroups:

1. Gym-based exercise program for obese individuals (OB-GYM; *n* = 49);
2. NW program for obese individuals (OB-NW; *n* = 37);
3. Gym-based exercise program for obese individuals with DM2 (DM2-GYM; *n* = 10);
4. NW program for obese individuals with DM2 (DM2-NW; *n* = 12).

The baseline characteristics of participants are shown in Table 1.

### 2.2. Study Design

The C.U.R.I.A.Mo. clinical model, previously described by De Feo [24], provides the participation of master-trained specialists, who work following a multidisciplinary method. The C.U.R.I.A.Mo. project has been registered in the Australian New Zealand Clinical Trials Registry (a Primary Registry in the WHO registry network), with the number: ACTRN12611000255987.

All the participants gave their written informed consent to participate in the study, and prior to starting any kind of assessments, were asked to fill out a questionnaire regarding the possible presence of musculoskeletal disorders, which could influence our choice when assigning the subjects to either the GYM or NW program. Using a quasi-experimental study design, individuals were assessed before (T0) and at the end of each exercise intervention (T1).

### 2.3. The C.U.R.I.A.Mo. Clinical Model

All the participants have been assessed through the C.U.R.I.A.Mo. evaluation model composed by four clinical steps.

1. A medical examination was managed by the endocrinologist to exclude the presence of clinical conditions that could contraindicate the exercise interventions. During the visit, the C.U.R.I.A.Mo. clinical model was explained to participants, and the blood tests were prescribed according to national standards of care [25].
2. A psychological interview to increase the subjects’ lifestyle change and to assess their psychological status.
3. A nutritional evaluation to assess the nutritional habits of the participants in order to increase their awareness of a balanced daily diet based on the Mediterranean dietary principles. The C.U.R.I.A.Mo. model provides two individual counseling sessions (before and after the interventions). Nutritional counseling aims to reduce saturated and trans-unsaturated fatty acids to under 10% of the total daily energy, promoting the consumption of fish, vegetables, legumes, fruit and whole grain cereals, to reduce calorie intake. Participants were also invited to attend educational classes focused on healthy diets and good physical activity habits.
4. A complete medical examination was performed by a physician focused on assessing the individual aerobic capacity and muscle strength, and to exclude any potential contraindications to exercise. These outcomes were also used to increase the participants’ awareness of their individual physical status.

### 2.4. Exercise Interventions

The two forms of supervised exercise programs (NW and GYM) were planned in accordance with the main international guidelines on exercise prescriptions (American College of Sport Medicine, ACSM) and other previous studies [7,26,27,28]. Both indoor (GYM) and outdoor (NW) programs were supervised by a specialist sports science graduate. Blood pressure and blood glucose values were recorded at the beginning and at the end of each training session. All the participants were constantly monitored through a heart rate (HR) monitor to ensure that the training program was performed according to the target intensity suggested by ACSM [26]. Furthermore, adherence to each exercise program was recorded and calculated. Specific reports about the two interventions are presented in Table 2.

#### 2.4.1. Nordic Walking Program

The Nordic walking program was performed on a 750 m-long circular path that was located in the natural park area of the Bambagioni Sporting Centre at the University of Perugia. Exercise sessions were performed 2 times per week and lasted 90 min (10 min of warm up, 60–65 min of NW, and 15 min of cool down). The training intensity was gradually increased, starting from 40% up to 60% of the HR reserve (calculated using the Karvonen formula [29]), through gradual steps of increment. Before the training program, 2 weeks were dedicated to a familiarization with the NW technique.

#### 2.4.2. GYM Program

The gym-based exercise program was planned as a combined circuit training protocol, performed 2 times per week, and lasted 90 min. Before and after the circuit training protocol, 10 min of warm up and 15 min of cool down aerobic exercise (walking, or treadmill, or cycling) were performed. The main part of the sessions consisted of a set of exercises involving the large muscle groups of the lower and upper limbs; the circuit training mixed aerobic and strength exercise, alternating six aerobic exercises (e.g., cycling and walking) and five strength exercise stations (e.g., leg press, abdominal exercises, pectoral exercises performed with isotonic machines and free weights). Aerobic exercise intensity was monitored and gradually increased, as in the NW protocol. In addition, strength training intensity increased gradually, starting from 55% of one repetition maximum (1-RM) and, in time, up to 70%. Brzycki equation was used to predict the 1-RM estimated value (1-RM = 100 × (load repetition, or workload value of repetition performance, expressed in kg)/(102.78 − 2.78 * number of repetitions performed)) [30].

### 2.5. Specific Functional and Clinical Assessments

Before starting and at the end of the two exercise interventions all participants underwent a clinical and functional assessment. Anthropometric parameters included waist circumference (WC), height, body weight (BW), body mass index (BMI), and waist–height ratio (WHR) evaluated using standard techniques [31,32,33]. Body composition (fat mass and fat-free mass indexes) was also evaluated through the use of the Tanita body composition analyzer BC-420MA (Tokyo, Japan) [34]. Systolic and diastolic blood pressure (BP) values were measured through a UM-101 mercury-free sphygmomanometer (A&D Medical, Tokyo, Japan) during the first ambulatory visit. Blood chemistry variables, such as fasting blood glucose, glycated hemoglobin (HbA1c), total cholesterol, high-density lipoprotein (HDL) cholesterol and triglycerides were collected in the laboratory analyses report. Fitness variables, such as aerobic fitness (maximal oxygen uptake, VO_2_ max) and muscular flexibility were evaluated using the Rockport fitness walking test [35] and the Bending test (executed from vertical and horizontal position) [36], respectively.

### 2.6. Statistical Analysis

An analysis of variance was performed to check any differences in baseline values (T0) among the 4 subgroups. Table 1 presented the mean values for the entire sample and for each group of exercise.

To evaluate the effects of the two forms of exercise programs, parameters at baseline and after three months of the exercise interventions were compared for each group through *t*-test for paired samples. Delta (Δ) changes (T1-T0) are presented as means and standard deviations (SDs).

Finally, to compare the NW and GYM exercise program effects, an analysis of variance of Δ changes (T1-T0) was performed. *p* values ≤ 0.05 were set as statistically significant. All the data have been digitally archived and the analyses were performed using SPSS^®^ Software, version 22.0 (IBM Corp. Released 2013. IBM SPSS Statistics for Windows, Version 22.0. Armonk, NY: IBM Corp).

## 3. Results

One hundred and eight obese subjects were involved. All of them completed the entire exercise program and no side effects or injury were recorded. The rate of participation to the training sessions was 19.90 ± 4.28 out of 24 total sessions. The general percentage of adherence to both the exercise programs was 80.68 ± 14.47. Moreover, higher percentage of adherence to the GYM (OB-GYM = 87.22 ± 9.45; DM2-GYM = 87.30 ± 10.35) compared to the NW program (OB-NW = 71.86 ± 14.82; DM2-NW = 75.58 ± 17.90) was observed.

**Figure 2:**
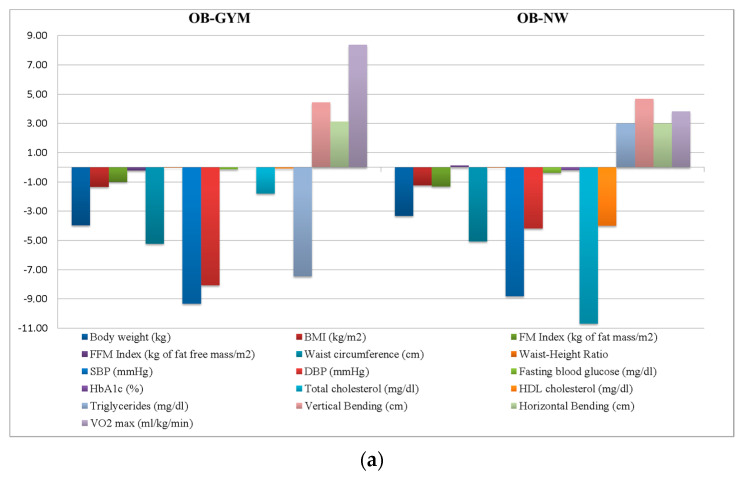

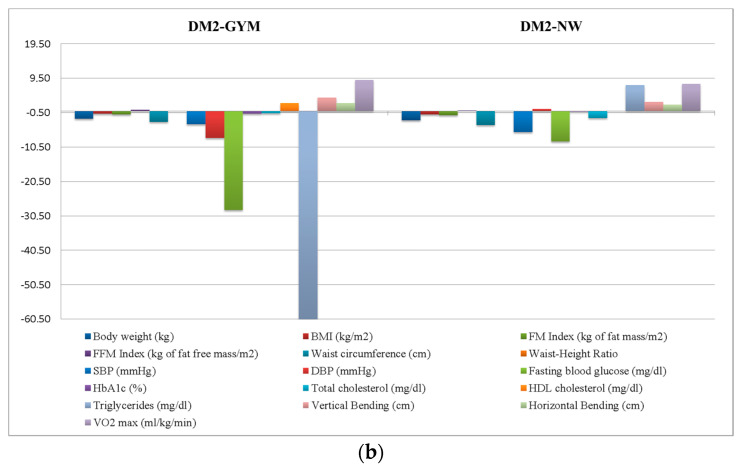
(**a**) Anthropometric profile, blood pressure levels, blood chemistry and fitness parameters in obese people. Results are presented as Δ (T1-T0) means. (**b**) Anthropometric profile, blood pressure levels, blood chemistry and fitness parameters in people with type 2 diabetes. Results are presented as Δ (T1-T0) means. Abbreviations: **OB-GYM**: individuals with obesity participating at gym-based exercise program; **OB-NW**: individuals with obesity participating at the Nordic walking exercise program; **DM2-GYM**: obese individuals with DM2 participating at the gym-based exercise program; **DM2-NW**: obese individuals with DM2 participating at the Nordic walking program.

In all of the four groups, a statistically significant reduction in BW, BMI, fat mass index, muscular flexibility in vertical position (vertical bending) and VO_2_ max were observed. For more information, please see: Figure 2a,b and Supplementary Material (attached file: “Table S1. Post-intervention assessments”).

In individuals with obesity only, significant improvements in BW, BMI, fat mass index, WC, WHR, vertical bending values, VO_2_ max (all, *p* < 0.01) and systolic BP levels (*p* = 0.01) were found at the end of both exercise programs. Notably, only the OB-NW group showed significant improvements in HbA1c values (*p* < 0.01), total and HDL cholesterol levels (*p* = 0.01).

In obese individuals with DM2, significant improvements in BW, BMI, fat mass index, vertical bending results and VO_2_ max levels were observed after both exercise programs. In addition, the DM2-GYM group showed significant improvements also in diastolic BP values (*p* = 0.01) and in the horizontal bending results (*p* = 0.04). In obese and diabetic individuals, the NW program allowed us to reach significant improvements also in WC and WHR (*p* ≤ 0.01) measurements. In the DM2-GYM group, we observed an important improvement in fasting blood glucose (−28.80 mg/dL) and triglycerides (−60.50 mg/dL). However, probably due to the small size of the sample, a significant statistic was not achieved. Overall, we found a significant relationship between proportional changes in plasma triglycerides and glucose metabolism after the program. In fact, proportional changes in plasma triglycerides explained 30% and 78% of the changes in plasma glucose and HbA1c, respectively. For more information please see Supplementary Materials (Table S2a and S2b).

## 4. Discussion

The aim of the study was to compare the cardiometabolic effects of two different supervised exercise programs (NW vs. GYM), following a multidisciplinary intervention performed at the C.U.R.I.A.Mo. center. Both exercise programs were developed in accordance with the ACSM guidelines for exercise testing and prescription in people with DM2 [28]. All participants completed the program and no side effects were recorded; this result suggested that, if the training program is developed following the guidelines, NW and GYM are two safe training modalities for obese patients with or without DM2 [37,38].

The promotion of general physical activity for health purposes, in the context of multidisciplinary lifestyle interventions, is essential to contrast sedentary lifestyle, which in turn contributes to an increase in the incidence of obesity and many other kinds of non-communicable chronic diseases [39]. Moreover, it was well-established that different forms of exercise-based interventions are strongly recommended in DM2 management [40]. In fact, the American Diabetes Association and the European Association for the Study of Diabetes stated that aerobic exercise, and the combination of aerobic exercise plus resistance training may be more effective than resistance training alone [41]. Structured and supervised exercise programs involving individuals with DM2 have been shown to be effective in antiatherogenic positive changes and in improving cardiometabolic parameters [5,42]. The C.U.R.I.A.Mo. multidisciplinary intervention already demonstrated encouraging results in obese subjects for some metabolic parameters [43], and in the glycemic control of those individuals with DM2 [44]. However, our findings reinforced the key role played by structured and supervised forms of exercise in DM2 management, underlining the therapeutic usefulness of non-conventional approaches, as is the case of NW training. Furthermore, our study compared two different forms of supervised exercise programs, providing an innovative message: in addition to the best-known conventional exercise-based therapies, special populations can choose among different types of validated approaches to exercise, according to their attitudes, motivations and interests.

As demonstrated by the findings of the UK Prospective Diabetes Study (UKPDS) [45], the positive changes in some cardiovascular risk factors and metabolic parameters resulted in a significant modification of the general cardiovascular risk score, crucial for increasing long-term cardiovascular protection. In obese and DM2 exercise groups, this study demonstrated the efficacy of both gym-based and NW exercise to improve BW, WC, BMI and fat mass index parameters. In this line, our findings show that in obese adults, a reduction in WHR measures was detected. Such element is closely connected with the decrease in general cardiovascular risk profile. Furthermore, the improvement in cardiorespiratory fitness (VO_2_ max values) has been shown to be associated with a reduced total and cardiovascular mortality [46], both in the general and in the DM2 population [47]. Indeed, some of the previous studies [48,49] reported that increases of approximately 3.5 mL/min/kg in VO_2_ max values are associated with a reduction in all-cause mortality superior to 10%. In our study, increments of VO_2_ max have shown to reach values superior to these data [48,49] (GYM groups: 8.38 and 9.06; NW groups: 3.83 and 7.9), supporting the strong need to comprise the therapeutic exercise within the multidisciplinary care of populations with metabolic diseases.

Although many studies showed that the exercise performed in a natural environment determines greater adherence [50,51,52,53], our findings registered an opposite result. According to other authors [54,55,56], it is possible that individuals, who participated at the NW program, being mainly women, meet multiple barriers to assure a continuative exercise participation, such as lack of time (e.g., due to household tasks) and feelings of guilt. Despite the lower adherence (−14.4%), the NW program determined the same positive responses to GYM in individuals with DM2. Furthermore, in the obese individuals NW determined a greater reduction in metabolic values. This could indicate that NW has a greater cardiometabolic efficacy than the GYM program, likely due to the continuous active use of muscles of trunk, upper and lower limbs.

In their study [57,58], Balducci et al. reported that there is not yet conclusive evidence that positive changes in physical activity levels can be sustained over the long term, however, our study highlighted that supervised exercise programs can support patients’ adherence to exercise-based treatment, recording a general activities participation of 80.7%. According to Italian [25] and ACSM/ADA [26] guidelines, our results support the recommendation for qualified exercise-trainer supervision (graduate sports science specialists), both to minimize risk of injuries and overall to achieve tailored health aims that are capable of effectively counteracting such kinds of non-communicable diseases from spreading worldwide. Other authors have shown that unsupervised NW trainings seem to not fulfill enough increase in exercise intensity in order to achieve those health advantages specific to individuals with DM2 [59]. From this point of view, for those subjects belonging to special populations who choose to carry out unconventional therapeutic exercise-based activities such as NW, aquatic exercise and other fitness workouts, it is imperative to follow the main guidelines on exercise prescription and be supported by the guidance of exercise science specialists for the entire duration of tailored exercise programs [60,61].

## 5. Conclusions

Our findings show that a supervised NW program is as effective as a conventional GYM program in improving BW control, body composition, muscular flexibility and VO_2_ max levels in obese adults with and without DM2; notwithstanding, we recorded a significantly lower adherence to NW than GYM programs. Furthermore, NW is an easy-to-learn activity performable in different environments, rarely associated with physical injuries, more adaptable than GYM with regard to lack of time, low fitness level, and shortage of money, requiring only specific poles. This allows us to state the major efficacy of NW over GYM program, in improving cardiometabolic parameters of both obese people with and without DM2. However, the lack of a normal-weight control group did not permit a deepened knowledge of the effects of NW and GYM programs on cardiometabolic parameters in obese subjects with and without DM2. Ultimately, our study emphasizes the awareness that in addition to the best-known conventional exercise-based therapies, populations with metabolic diseases can opt also to do other forms of validated exercises according to their attitudes, motivations and interests.

## Figures and Tables

**Table 1:** Anthropometric, blood pressure, blood chemistry, and fitness parameters at baseline**. Baseline values:** Mean values of anthropometric, blood pressure and blood chemistry parameters in the entire sample and in the 4 subgroups. Data are presented as means ± SDs. Statistical significance was set for *p* values ≤ 0.05.

Outcomes	Total Sample *n* = 108 (F = 73; M = 35)	OB-GYM *n* = 49 (F = 33; M = 16)	OB-NW *n* = 37 (F = 30; M = 7)	DM2-GYM *n* = 10 (F = 4; M = 6)	DM2-NW *n* = 12 (F = 6; M = 6)	F	*p* Values
**Age**	56.44 ± 5.94	55.29 ± 5.67	56.62 ± 5.8	59.6 ± 6.5	58 ± 6.35	1.89	0.14
**Body weight (kg)**	100.75 ± 15.4	101.07 ± 16.71	98.50 ± 14.87	104.51 ± 14.38	103.23 ± 12.58	0.57	0.64
**BMI (kg/m^2^)**	36.47 ± 5.11	36.6 ± 4.99	36.37 ± 5.85	35.48 ± 4.32	37.07 ± 4.07	0.19	0.90
**FM index (kg of fat mass/m^2^)**	15.27 ± 4.24	15.22 ± 3.91	15.74 ± 4.64	13.62 ± 4.16	15.46 ± 4.6	0.65	0.58
**FFM index (kg of fat free mass/m^2^)**	19.87 ± 2.27	20.05 ± 2.23	19.15 ± 1.99	20.77 ± 2.91	20.49 ± 2.36	2.17	0.08
**Waist circumference (cm)**	115.93 ± 10.52	114.88 ± 11.26	115.5 ± 9.83	118.5 ± 9.88	119.25 ± 10.22	0.77	0.51
**Waist–Height Ratio**	0.7 ± 0.06	0.69 ± 0.07	0.7 ± 0.07	0.69 ± 0.06	0.72 ± 0.07	0.49	0.69
**SBP (mmHg)**	135 ± 13.08	134.69 ± 10.43	135 ± 12.95	137 ± 16.02	134.55 ± 21.27	0.09	0.97
**DBP (mmHg)**	82.52 ± 8.94	83.64 ± 7.83	80.86 ± 9.66	85 ± 7.07	80.46 ± 12.14	1.13	0.34
**Fasting blood glucose (mg/dL)**	105.13 ± 30.83	95 ± 12.64	94.62 ± 11.66	149 ± 58.25	138.83 ± 35.45	24.58	<0.01
**HbA1c (%)**	6.04 ± 0.99	5.71 ± 0.52	5.76 ± 0.41	7.17 ± 1.91	7 ± 1.07	15.17	<0.01
**Total cholesterol (mg/dL)**	210.76 ± 36.78	218.06 ± 35.26	207.91 ± 35.67	201.9 ± 41.52	196.67 ± 39.9	1.52	0.22
**HDL cholesterol (mg/dL)**	49.65 ± 11.48	49.83 ± 9.99	53.06 ± 13.86	43.1 ± 8.89	44.75 ± 8.07	2.98	0.04
**Triglycerides (mg/dL)**	151.81 ± 82.19	142.49 ± 66.51	130.67 ± 46.22	253.9 ± 169.79	162.92 ± 53.07	7.28	<0.01
**Vertical bending (cm)**	−9.67 ± 9.34	−9.89 ± 9.31	−6 ± 2	−12 ± 10.61	−8.6 ± 13.33	0.51	0.68
**Horizontal bending (cm)**	24.89 ± 9.72	24.26 ± 8.83	33.33 ± 7.06	21 ± 9.53	27 ± 15.68	2.26	0.09
**VO_2_ max (mL/kg/min)**	13.98 ± 9.25	14.40 ± 8.77	11.88 ± 12.85	14.77 ± 12	11.6 ± 2.85	0.16	0.92

Abbreviations: F: females; M: males; OB-GYM: individuals with obesity participating at gym-based exercise program; OB-NW: individuals with obesity participating in the Nordic walking exercise program; DM2-GYM: obese individuals with DM2 participating in the gym-based exercise program; DM2-NW: obese individuals with DM2 participating in the Nordic walking program; BMI: body mass index; FM: fat mass; FMM: fat-free mass; SBP: systolic blood pressure; DBP: diastolic blood pressure; HbA1c: glycosylated hemoglobin; HDL: high-density lipoprotein; VO_2_ max: maximal oxygen.

**Table 2:** Exercises for both Nordic walking (NW) program and gym-based exercise program (GYM).

Nordic Walking					
Phase	Exercise	Duration	Sets	Repetitions	Intensity
Warm up		10 min			
Main part	Nordic walking	60–65 min			40 to 60% HRR
Cool down	Stretching	15 min			
GYM Program					
Warm up	Treadmill, or walking, or cycling	10 min			
Main part	Treadmill	12 min			40 to 60% HRR
	Leg press		2	20	55 to 70% 1-RM
	Cycle ergometer	4 min			40 to 60% HRR
	Lat machine		2	20	55 to 70% 1-RM
	Arm ergometer	4 min			40 to 60% HRR
	Chest press		2	20	55 to 70% 1-RM
	Cardio	4 min			40 to 60% HRR
	Abdominal		3	10–15	55 to 70% 1-RM
	Cardio	4 min			40 to 60% HRR
	Leg extension		2	20	55 to 70% 1-RM
Cool down	Treadmill, or walking, or cycling Stretching	15 min			

Abbreviations: HRR: heart rate reserve; 1RM: 1-repetition maximum.

## Supplementary Materials

The following are available online at https://www.mdpi.com/2411-5142/5/3/62/s1, Table S1: Post-intervention assessments; Table S2a and S2b: Descriptive statistics and Delta changes of plasma triglycerides and glucose metabolism correlations.
