# Supplementary material for: Effects of a Supervised Nordic Walking Program on Obese Adults with and without Type 2 Diabetes: The C.U.R.I.A.Mo. Centre Experience
Source: J Funct Morphol Kinesiol. 2020 Aug 7;5(3):62. doi: 10.3390/jfmk5030062 (PMC7739339; doi:10.3390/jfmk5030062)
Supplement: Supplementary file 1 [file jfmk-05-00062-s001.pdf]

Table S1. Post-intervention assessments: Anthropometric profile, blood pressure levels, blood chemistry and fitness parameters in the 4 subgroups. Results are presented as Δ (T1-T0), means and SDs. Statistical significance was set for *p* values ≤.05.

	**OB-GYM** *n=49*				**OB-NW** *n=37*				**DM2-GYM** *n=10*				**DM2-NW** *n=12*				**Between-groups factor**	
	Δ Mean	*SD*	*t*	*p*	*Δ Mean*	*SD*	*t*	*p*	*Δ Mean*	*SD*	*t*	*p*	*Δ Mean*	*SD*	*t*	*p*	*F*	*p*
**Body weight (kg)**	-3.98	4.28	-6.50	<.01	-3.32	4.12	-4.91	<.01	-2.20	3.12	-2.23	.05	-2.75	3.47	-2.75	.02	0.72	.54
**BMI (kg/m²)**	-1.35	1.66	-5.71	<.01	-1.24	1.62	-4,65	<.01	-0.75	1.06	-2.23	.05	-1.03	1.28	-2.77	.02	0.48	.70
**FM Index (kg of fat mass/m²)**	-1.00	1.43	-4.85	<.01	-1.33	1.85	-4.24	<.01	-1.00	1.02	-3.08	.01	-1.23	1.29	-3.16	.01	0.35	.79
**FFM Index (kg of fat free mass/m²)**	-0.21	0.96	-1.53	.13	.13	1.14	0.67	.51	0.34	1.40	0.77	.46	0.03	0.78	0.15	.89	1.17	.33
**Waist circumference (cm)**	-5.22	5.62	-6.29	<.01	-5.06	5.55	-5.08	<.01	-3.10	5.00	-1.96	.08	-4.11	2.57	-4.80	<.01	0.21	.89
**Waist-Height Ratio**	-0.03	0.03	-6.41	<.01	-0.03	0.03	-5.09	<.01	-0.02	0.03	-1.90	.09	-0.02	0.01	-5.05	<.01	0.65	.58
**SBP (mmHg)**	-9.32	13.01	-4.75	<.01	-8.81	17.17	-2.86	.01	-3.89	11.40	-1.02	.34	-6.11	14.53	-1.26	.24	0.43	.73
**DBP (mmHg)**	-8.07	11.67	-4.58	<.01	-4.19	15.28	-1.53	.14	-7.78	6.67	-3.50	.01	0.56	8.46	0.20	.85	1.51	.22
**Fasting blood glucose (mg/dl)**	-0.13	11.91	-0.07	.94	-0.39	9.37	-0.23	.82	-28.80	64.47	-1.41	.19	-8.89	23.70	-1.13	.29	4.49	.01
**HbA1c (%)**	0.005	0.32	0.10	.92	-0.20	0.22	-4.62	<.01	-0.77	1.65	-1.48	.17	-0.23	0.79	-0.92	.38	3.79	.01
**Total cholesterol (mg/dl)**	-1.81	26.87	-0.47	.64	-10.69	21.76	-2.78	.01	-0.70	26.34	-0.08	.93	-2.00	35.67	-0.18	.86	0.86	.47

**HDL cholesterol (mg/dl)**	-0.09	8.84	-0.07	.95	-4.00	7.43	-2.95	.01	2.30	8.23	0.88	.40	0.00	5.91	0.00	1.00	2.17	.10
**Triglycerides (mg/dl)**	-7.45	46.73	-1.09	.28	3.00	49.50	0.32	.75	-60.50	135.70	-1.41	.19	7.50	59.54	0.40	.70	2.78	.05
**Vertical Bending (cm)**	4.45	6.79	4.25	<.01	4.67	2.73	4.18	.01	3.89	3.41	3.42	.01	2.60	1.34	4.33	.01	0.13	.94
**Horizontal Bending (cm)**	3.14	4.89	4.16	<.01	3.00	3.67	1.83	.14	2.33	2.78	2.51	.04	1.80	4.55	0.88	.43	0.30	.82
**VO_2 max (ml/kg/min)**	8.38	6.40	8.59	<.01	3.83	3.65	2.57	.05	9.06	6.40	4.25	<.01	7.9	3.06	5.77	<.01	5.70	<.01

Abbreviations: **OB-GYM**: individuals with obesity participating at gym-based exercise program; **OB-NW**: individuals with obesity participating at the Nordic walking exercise program; **DM2-GYM**: obese individuals with DM2 participating at the gym-based exercise program; **DM2-NW**: obese individuals with DM2 participating at the Nordic walking program; **BMI:** body mass index; **FM**: fat mass; **FFM**: fat free mass; **SBP**: systolic blood pressure; **DBP**: diastolic blood pressure; **HbA1c:** glycosylated haemoglobin; **HDL**: high-density lipoprotein; **VO_2 max**: maximal oxygen uptake.

Table S2a. Descriptive statistics: Results are presented as Δ (T1-T0) means and Standard Deviation (SDs).

	Mean	SDs
ΔGLI	-3,237	25,8371
ΔHBA1C	-,174	,6867
ΔCOL	-4,560	26,2241
ΔHDL	-1,041	8,2600
ΔTG	-8,379	65,0380

Abbreviations: **GLI:** Fasting blood glucose; **HbA1c:** glycosylated haemoglobin; **COL:** Total cholesterol**; HDL**: high-density lipoprotein; **TG**: Triglycerides.

<u>Table S2b</u>. Delta changes of plasma triglycerides and glucose metabolism correlations.

		ΔGLI	ΔHBA1C	ΔCOL	ΔHDL	ΔTG
ΔGLI	Pearson Correlations					
	Sign. (two-tailed)					
ΔHBA1C	Pearson Correlations	0,781**				
	Sign. (two-tailed)	0,000				
ΔCOL	Pearson Correlations	0,108	-0,023			
	Sign. (two-tailed)	0,299	0,833			
ΔHDL	Pearson Correlations	0,124	0,192	0,069		
	Sign. (two-tailed)	0,240	0,084	0,505		
ΔTG	Pearson Correlations	0,545**	0,474**	0,175	-0,187	
	Sign. (two-tailed)	0,000	0,000	0,089	0,071	

<u>Abbreviations:</u> **GLI:** Fasting blood glucose; **HbA1c:** glycosylated haemoglobin; **COL:** Total cholesterol**; HDL**: high-density lipoprotein; **TG**: Triglycerides.

Statistical significance was set for *p* values ≤.05
